# International Consortium to Classify Ageing-related Pathologies (ICCARP) senescence definitions: achieving international consensus

**DOI:** 10.1007/s11357-025-01509-9

**Published:** 2025-02-21

**Authors:** Emma Short, Robert T. R. Huckstepp, Kambiz Alavian, Winfried M. K. Amoaku, Thomas M. Barber, Edwin J. R. van Beek, Emyr Benbow, Sunil Bhandari, Phillip Bloom, Carlo Cota, Paul Chazot, Gary Christopher, Marco Demaria, Jorge D. Erusalimsky, David A. Ferenbach, Thomas Foster, Gus Gazzard, Richard Glassock, Noordin Jamal, Raj Kalaria, Venkateswarlu Kanamarlapudi, Adnan H. Khan, Yamini Krishna, Christiaan Leeuwenburgh, Ian van der Linde, Antonello Lorenzini, Andrea Britta Maier, Reinhold J. Medina, Cecilia L. Miotto, Abhik Mukherjee, Krishna Mukkanna, James T. Murray, Alexander Nirenberg, Donald B. Palmer, Graham Pawelec, Venkat Reddy, Arianna Caroline Rosa, Andrew D. Rule, Paul G. Shiels, Carl Sheridan, Jeremy Tree, Dialechti Tsimpida, Zoe C. Venables, Jack Wellington, Stuart R. G. Calimport, Barry L. Bentley

**Affiliations:** 1 Cardiff School of Technologies, Cardiff Metropolitan University, Cardiff, UK; 2 Department of Cellular Pathology, Swansea Bay University Health Board, Swansea, UK; 3 School of Life Sciences, University of Warwick, Coventry, UK; 4 Department of Brain Sciences, Faculty of Medicine, Imperial College London, London, UK; 5 School of Medicine, University of Nottingham, Nottingham, UK; 6 Warwickshire Institute for the Study of Diabetes, Endocrinology and Metabolism, University Hospitals Coventry and Warwickshire, Clifford Bridge Road, Coventry, UK; 7 Division of Biomedical Sciences, Warwick Medical School, University of Warwick, Coventry, UK; 8 Edinburgh Imaging Facility, Queen’s Medical Research Institute, Edinburgh, UK; 9 NHS Lothian Health Board, Edinburgh, UK; 10 Manchester Medical School, University of Manchester, Manchester, UK; 11 Hull Teaching Hospitals NHS Trust, Hull, UK; 12 Hull York Medical School, Hull, UK; 13 Imperial College Healthcare NHS Trust, London, UK; 14 Genetic Research, Molecular Biology and Dermatopathology Unit, San Gallicano Dermatological Institute, IRCCS, Rome, Italy; 15 Durham University, Durham, UK; 16 Centre for Ageing and Dementia Research, Swansea University, Swansea, UK; 17 European Research Institute for the Biology of Ageing, Groningen, the Netherlands; 18 Institute for Mechanisms of Health, Ageing and Disease (MoHAD), University Medical Center Groningen, Groningen, the Netherlands; 19 The Cellular and Molecular Pathophysiology Group, Cardiff Metropolitan University, Cardiff, UK; 20 Centre for Inflammation Research, Institute for Regeneration and Repair, University of Edinburgh, Edinburgh, UK; 21 University of Florida, Gainesville, FL USA; 22 Moorfields Eye Hospital NHS Foundation Trust, London, UK; 23 UCL Institute of Ophthalmology, London, UK; 24 Department of Medicine, Geffen School of Medicine at UCLA, Los Angeles, CA USA; 25 University of East Anglia, Norwich, UK; 26 Translational and Clinical Research Institute, Newcastle University, Newcastle Upon Tyne, UK; 27 Swansea University Medical School, Swansea University, Swansea, UK; 28 Clinical and Experimental Sciences, Faculty of Medicine, University of Southampton, Southampton, UK; 29 Liverpool Clinical Laboratories, National Specialist Ophthalmic Pathology Service, Liverpool University Hospitals NHS Foundation Trust, Liverpool, UK; 30 Department of Eye and Vision Science, Institute of Life Course and Medical Science, University of Liverpool, Liverpool, UK; 31 Department of Physiology and Aging, College of Medicine, University of Florida, Gainesville, FL USA; 32 Cognition and Neuroscience Group, ARU Centre for Mind and Behaviour, Faculty of Science & Engineering, Anglia Ruskin University, Cambridge, UK; 33 Department of Biomedical and Neuromotor Sciences, University of Bologna, Bologna, Italy; 34 National Institute of Biosystems and Biostructures INBB, Rome, Italy; 35 NUS Academy for Healthy Longevity, Yong Loo Lin School of Medicine, National University of Singapore, Singapore, 117597 Singapore; 36 Department of Human Movement Sciences, Faculty of Behavioural and Movement Sciences, Amsterdam Movement Sciences, Vrije Universiteit Amsterdam, 1081 BT Amsterdam, the Netherlands; 37 Translational Medical Sciences, School of Medicine, University of Nottingham, Nottingham, UK; 38 University Hospital of Wales, Cardiff, Wales UK; 39 Cardiff and Vale University Health Board, Cardiff, UK; 40 Australasian College of Cutaneous Oncology, Docklands, Australia; 41 Dorevitch Pathology, Heidelberg West, Australia; 42 Department of Comparative Biomedical Sciences, Royal Veterinary College, University of London, London, UK; 43 Department of Immunology, University of Tübingen, Tübingen, Germany; 44 Health Sciences North Research Institute, Sudbury, ON Canada; 45 Department of Ageing, Rheumatology and Regenerative Medicine, Division of Medicine, University College London, London, UK; 46 Department of Rheumatology, University College Hospital, London, UK; 47 Department of Drug Science and Technology, University of Turin, Turin, Italy; 48 Departments of Medicine and of Quantitative Health Sciences, Mayo Clinic, Rochester, USA; 49 Glasgow Geroscience Group, School of Molecular Biosciences, MVLS, University of Glasgow, Glasgow, UK; 50 Director of the Advanced Diagnostics and Medical Technologies Research Institute, Faculty of Medicine, Health and Life Science, Swansea University, Swansea, UK; 51 Centre for Research On Ageing, Department of Gerontology, University of Southampton, Southampton, UK; 52 Norfolk and Norwich University Hospital, Norwich, UK; 53 Norwich Medical School, Norwich, UK; 54 Leeds Teaching Hospitals NHS Foundation Trust, Leeds, UK; 55 Collaboration for the Advancement of Sustainable Medical Innovation (CASMI), University College London, London, UK; 56 Center for Engineering in Medicine and Surgery, Harvard Medical School, Boston, MA USA; 57 Department of Surgery, Massachusetts General Hospital, Harvard Medical School, Boston, MA USA; 58 Shriners Children’s, Boston, MA USA

Dear Editors,

## Senescence definitions: ICCARP consensus

With the global increase in ageing populations, a clear understanding of the physiological and pathological changes associated with ageing is vital for advancing research and clinical practice. Following the World Health Organization’s decision to classify age-related aetiologies [1], the International Consortium to Classify Ageing-related Pathologies (ICCARP) was established in 2023, led by Cardiff Metropolitan University [2].

The aim of the ICCARP is to develop a systematic and comprehensive classification system for ageing-related changes including pathologies, diseases, and syndromes. Currently, the ICCARP is in the process of identifying all phenomena that meet the criteria for ageing-related pathologies, to develop proposals for grouping and naming them within a comprehensive classification system. However, during the course of this project, it became evident that certain terms, specifically relating to ‘senescence’, were interpreted and understood in multiple ways, often dependent upon the professional background of an expert and the context in which the term was being used. To achieve our goals, it is vital that we use a universal language when naming and proposing ageing-related changes to provide a clear, unambiguous understanding of the changes and their underlying contribution to maintaining or degrading organismal integrity (physiology versus pathology). Furthermore, establishing clear nomenclature will be advantageous in the wider efforts to unify the study of ageing, and to better align research and clinical practice.

The purpose of this letter is to explicitly state the definitions primarily relating to ‘senescence’ that will be used by the ICCARP, as agreed by ICCARP members through consensus meetings in 2024. The terms that will be defined are as follows:

1. Normative ageing
2. Senescence
3. Cellular senescence, including acute and chronic senescence
4. Physiological senescence
5. Pathological senescence
6. Tissue senescence
7. Organ senescence
8. Systems senescence
9. Organismal senescence

## Definitions

### Normative ageing

**Fig. 1:**
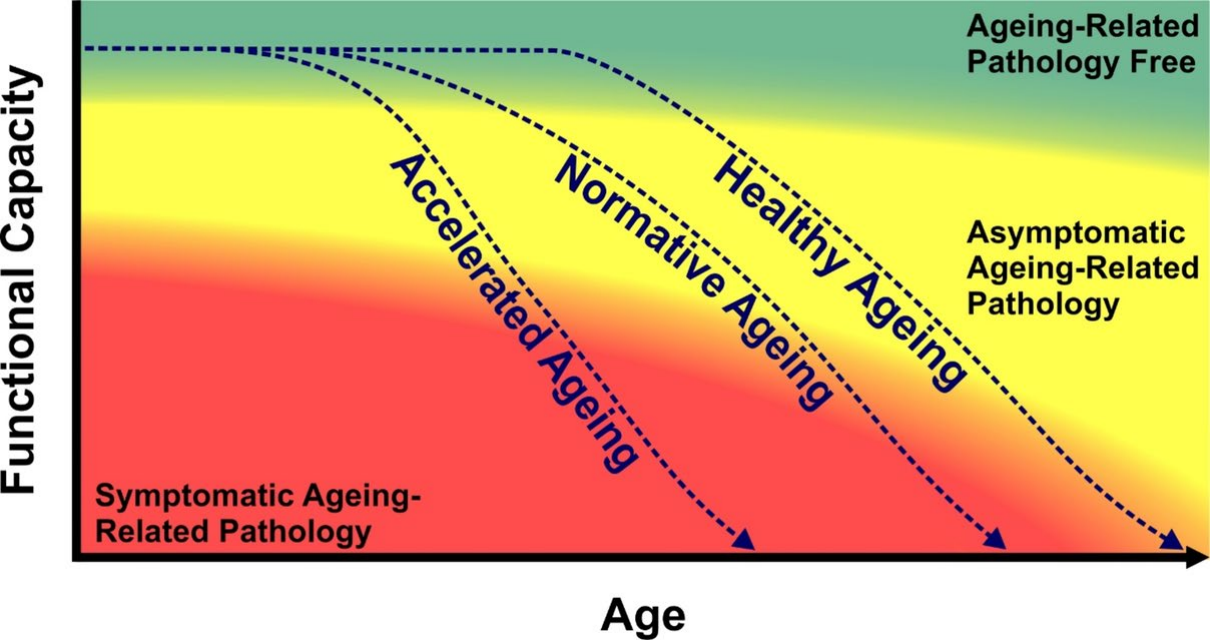
Conceptual diagram of senescence trajectories through ageing-related pathologies as function declines with chronological age. Note: the ‘Normative Ageing’ trajectory represents the experience of the average individual within a population, with ‘Healthy Ageing’ representing a possible trajectory of individuals with protective factors that decrease the rate of senescence, and ‘accelerated Ageing’ representing a possible trajectory of individuals with risk factors that accelerate the rate of ageing. ‘Ageing-related pathology free’ is a state with no increased risk of ageing-related morbidity or ageing-related premature mortality. ‘Asymptomatic Ageing-Related Pathology’ is a state with no symptoms but which is associated with an increased risk of progression to ageing-related morbidity or ageing-related premature mortality. ‘Symptomatic ageing-related pathology’ is a symptomatic state of increased risk of ageing-related morbidity or ageing-related premature mortality

Every individual ages uniquely. Whilst the literature has used terms such as ‘average’ and ‘typical’ ageing, we as a consortium believe the term *normative ageing*, defined as *the expected trajectory of ageing based on data derived from a particular population*, best encapsulates this phenomenon (Fig. 1). For the purposes of the identification, characterisation, and classification of ageing-related pathologies, ‘normative ageing’ is understood to encompass ageing-related cellular, tissue, organ, system or organismal senescence (defined below), and may include certain degrees of functional decline.

### Senescence

At the broadest possible level, it was agreed that *senescence* should describe a decline in normal functioning that occurs with chronological age (Fig. 1). Throughout human history, ageing-related changes, particularly those that are universally experienced, have been viewed differently from disease. Ageing-related changes influence patient expectations of clinical care and societal expectations for work and service. They are intrinsically linked to the human lifespan and are the result of a complex interplay between genetic, environmental, and lifestyle factors that influence the body’s ability to maintain and repair itself.

### Cellular senescence, including acute and chronic senescence

*Cellular senescence* is a state of indefinite cell cycle arrest that arises as a consequence of exhaustive cell proliferation (i.e., replicative senescence) or various stressors, including exposure to genotoxic and oxidative agents, sustained nutrient deprivation, and oncogene activation. Growth arrest is mediated by several cyclin-dependent kinase (CDK) inhibitors, primarily p16 and p21 [3, 4].

In addition to the generally irreversible growth arrest defining cellular senescence, senescent cells are commonly characterised by distinctive features such as altered morphology, telomere degradation or other macromolecular damage, deregulated metabolism, and a heterogeneous and context-dependent hypersecretory phenotype, known as the senescence-associated secretory phenotype (SASP). These morphological, structural, and functional changes distinguish senescent cells from G0 quiescent or terminally differentiated cells.

**Fig. 2:**
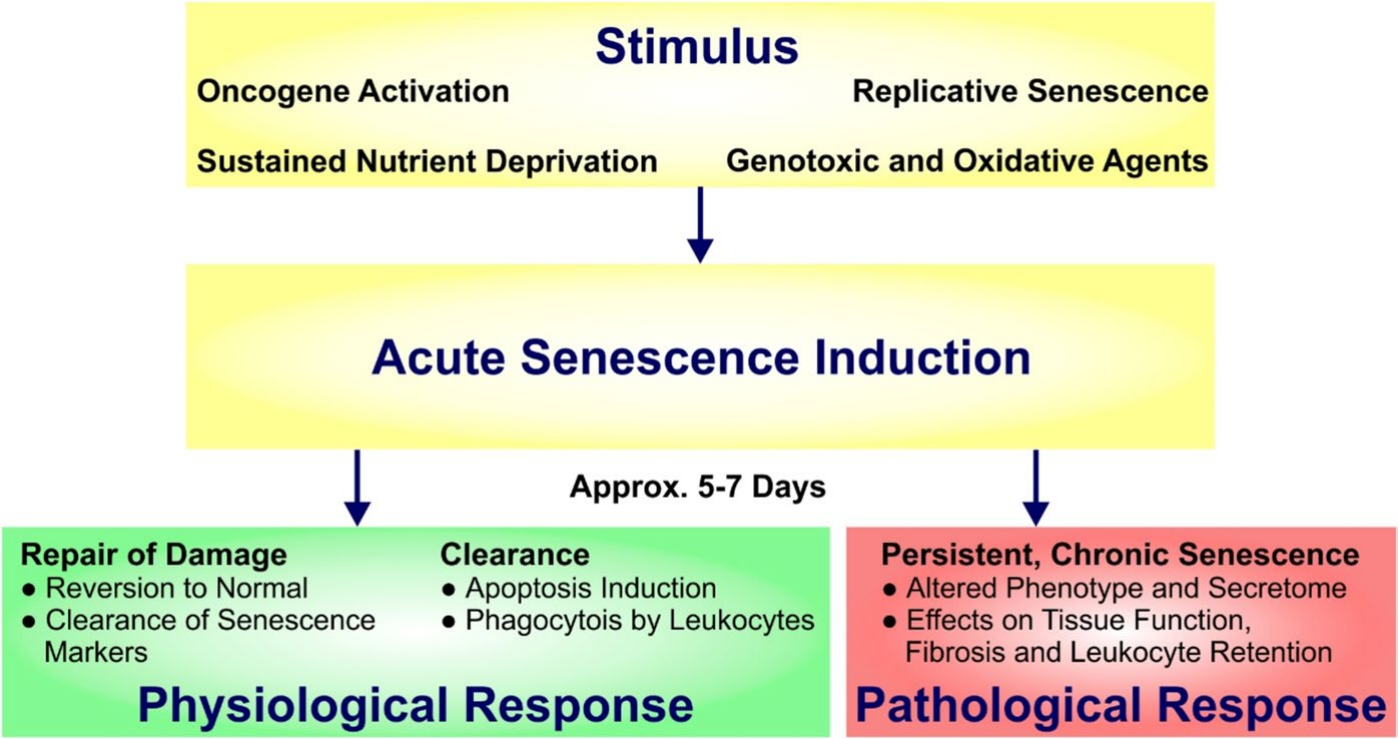
Acute senescence can occur due to a range of inciting factors and is typically short-lived. If senescent cells persist, chronic senescence may have pathological effects on an organism

Senescent cells can be detected at all life stages of an organism. *Acute senescence* is typically physiological and has many essential functions during development and tissue repair. In this context, it is important to note that senescent cells promote their own elimination by immune cells to maintain tissue integrity [5–7] (Fig. 2).

However, when senescent cells accumulate and persist in organs, in what is described as *chronic senescence*, this is associated with deteriorating organ function and contributes to ageing-related pathologies [8] (Fig. 2). As such, cellular senescence may be physiological or pathological.

### Physiological senescence

*Physiological senescence* is considered to be appropriate and necessary for the healthy functioning of an organism [9]. For example, it may be involved in processes such as development, tissue repair, tissue regeneration, maintaining tissue homeostasis, tissue remodelling, and potentially preventing tumour development in the early stages of an organism [9–11]. Physiological senescence is typically a short-term or acute process, as it has a defined purpose, and once that purpose has been achieved, the process ceases. However, in specific contexts, such as sustained tumour suppression, physiological senescence may occur over a longer period.

### Pathological senescence

*Pathological senescence* is defined as a process that causes a decline or deviation in function and homeostasis, that may be associated with structural changes. It refers to dysfunction or dysregulation of physiological processes across all manifestations of senescence—cellular, tissue, organ, system, and organismal—that may lead to the onset or progression of a disease, disorder, or syndrome. Pathological senescence may occur due to intrinsic factors, extrinsic factors, or a combination of both. Intrinsic factors include genetic mutations and epigenetic alterations, while extrinsic factors may include environmental stressors such as chronic inflammation, toxin exposure, or injury. Pathological senescence may be characterised by the persistent accumulation of senescent cells, impaired tissue regeneration, chronic inflammation, fibrosis, stem cell exhaustion, cell loss, an altered microenvironment, or tumourigenesis [9, 12–18].

An example of pathological senescence is seen in renal dysfunction, where p21 + senescent epithelial cells in the kidneys are implicated in reducing renal regenerative capacity with ageing and after injury, leading to increased levels of tissue fibrosis with loss of glomerular filtration rate in response to subsequent injuries [19]. Multiple molecular pathways, including C5a, DNA methylation, activation of Wnt4–β-catenin signalling, *Wnt9a* overexpression, inhibition of AMPK–mTOR signalling, and reactive oxygen species (ROS) have all been proposed to play a role in this [10, 20, 21].

### Tissue senescence

A *tissue* describes a collection of cells and their extracellular matrix, that are mutually organised to perform a specialised function, such as adipose tissue or cardiac muscle.

*Tissue senescence* is defined as an ageing-related decline in the functional capacity or structure of a tissue. This may result from the accumulation of senescent cells or be attributed to multiple underlying mechanisms. These include but are not limited to altered cell communication, genomic instability, aberrations in proteostasis, mitochondrial dysfunction, or changes in the extracellular matrix.

### Organ senescence

An *organ* refers to groups of mutually organised tissues that work together to perform a specific function. For example, the heart is composed of the endocardium, myocardium, epicardium, valves, vessels, and nerves.

*Organ senescence* describes an ageing-related decline in the functional capacity or structure of an organ, which can occur due to senescence of the tissues from which it is composed. This may be pathological, resulting in clinical sequelae, for example, cognitive decline due to brain senescence. It may also be an adaptation; for example, cardiac hypertrophy can be a compensatory mechanism to overcome systemic hypertension, but it increases the risk of heart failure. Organ senescence can also be normative, such as post-menopausal uterine senescence.

### Systems senescence

A *system* is a constellation of soluble factors, cells, tissues, or organs that work together to perform a specific function. For example, the immune system involves the integrated functions of soluble mediators such as cytokines, cells such as lymphocytes, tissues such as bone marrow, and organs such as the spleen, all of which can—either individually or collectively—manifest age-related functional impairments resulting in pathology. Systems typically work in conjunction with other systems.

*Systems senescence* refers to an ageing-related decline in the functional capacity of a biological system that may arise as a result of cellular, tissue, or organ senescence.

### Organismal senescence

An *organism* is the total of cells, tissues, organs, and systems that are structurally and functionally integrated to give rise to a cohesive living being. *Organismal senescence* refers to the gradual decline in physiological function and biological integrity that occurs as organism ages, resulting in a diminished ability to maintain homeostasis, repair damage, and respond to stressors. The process manifests in various ways across different species, and comparisons across vertebrate species show there are extremes of ageing phenotypes, ranging from atypically short to exceptionally long lifespans [22]. In humans, generally, organismal senescence involves a progressive deterioration in health, increased susceptibility to disease, and diminished reproductive capabilities. It is also intrinsically related to the lifespan of an organism.

Organismal senescence results from cellular, tissue, organ, or systems senescence and therefore, the hallmarks of organismal senescence should be defined by these parameters [23]. Broadly, organismal senescence should be considered as the consequence of changes that lead to a partial or complete loss of function at the organismal level.

## Conclusion

It is paramount that the language used in the scientific and medical literature is clear and unambiguous to ensure shared understanding amongst researchers, clinicians, and policymakers. We hope that this letter will serve as a reference to improve communication with respect to ageing and ensure clarity in future publications by the ICCARP, which will detail comprehensive and systematic classifications for ageing-related changes and pathologies. With a globally ageing population, standardised terminology related to ageing will be essential for enabling mutual understanding, and we expect that these definitions, and subsequent classifications, will contribute to improving discourse, research methodologies, clinical diagnostics, and public health planning.
